# Methicillin-Resistant Staphylococcus aureus Colonization in Intensive Care and Burn Units: A Narrative Review

**DOI:** 10.7759/cureus.47139

**Published:** 2023-10-16

**Authors:** Peter Samuel, Yash Sailesh Kumar, Bennet James Suthakar, Janadi Karawita, Divya Sunil Kumar, V Vedha, Heeya Shah, Keval Thakkar

**Affiliations:** 1 International Faculty of Medicine, Tbilisi State Medical University, Tbilisi, GEO; 2 Faculty of Medicine, Tbilisi State Medical University, Tbilisi, GEO; 3 Microbiology, Madras Christian College, Chennai, IND; 4 Internal Medicine, Medical University of South Carolina, Lancaster, USA; 5 Kidney Transplant, MedStar Georgetown University Hospital, Washington, USA

**Keywords:** management and prevention strategies, burn trauma, intensive care unit, cost effectiveness analysis, methicillin resistant staphylococcus aureus (mrsa)

## Abstract

Methicillin-resistant *Staphylococcus aureus* (MRSA) is a common hospital-acquired pathogen and can cause a wide spectrum of infections. In recent years, MRSA has emerged as a significant public health concern, particularly in hospitals. Intensive care units (ICUs) and burn units are high-risk areas for hospital-acquired MRSA infections, which can lead to increased morbidity, mortality, and healthcare costs. MRSA exhibits resistance to multiple antibiotics and can cause serious infections, including but not limited to pneumonia, endocarditis, and cutaneous infections, particularly in patients with burn injuries.

The prevention and effective management of MRSA infections in both burn patients and those in ICUs is crucial, with strategies like isolation, regular disinfection, and prophylactic intranasal mupirocin. Early diagnosis of MRSA infection and isolation of patients is vital to prevent the spread of MRSA. Implementation of prevention strategies faces many challenges, such as cost, and the most successful infection management practices are still debated.

This review has highlighted the substantial concern of MRSA colonization in intensive care and burn units. MRSA poses a significant risk to vulnerable patients, influenced by factors such as compromised immunity and invasive procedures. The prevalence of MRSA colonization varies, influenced by regional factors and infection control practices. Combating MRSA requires a multifaceted approach, including stringent infection control measures and education for healthcare workers and patients. As we move forward, continued research and cooperation are essential to reduce the burden of MRSA in these critical care settings.

## Introduction and background

*Staphylococcus aureus*, a gram-positive bacterium with a cocci-shaped morphology arranged in characteristic clusters resembling grapes, exhibits a positive gram-stain reaction. It is commonly present on most individuals' skin and mucous membranes, primarily inhabiting the nasal region. The bacterium can also be found in the typical human flora and various environmental niches. In instances where *S. aureus *gains access to the bloodstream or internal tissues, typically uncommon on healthy skin, it possesses the potential to instigate a range of diseases of significant severity. Direct contact is the major way the bacterium spreads, but some illnesses have other methods of transmission [1]. Additionally, we have retrieved 68 papers from PubMed, Directory of Open Access Journals, ScienceDirect, and Google Scholar.

Methicillin resistance in *S. aureus* is defined as an oxacillin minimum inhibitory concentration (MIC) of greater than or equal to 4 micrograms/mL based on antibiotic susceptibilities [2]. Hospital methicillin-resistant *Staphylococcus aureus* (MRSA) infections are more frequent in intensive care units (ICUs) and burn units, leading to higher healthcare costs and increased morbidity and mortality. The World Health Organization identified MRSA as one of the top three bacteria responsible for hospital infections [3]. MRSA poses a substantial concern because of its resistance to antibiotics, capacity to induce severe infections, ease of transmission in both healthcare and community environments, elevated healthcare expenses associated with treatment, and the formidable hurdles it presents in terms of infection control and prevention. It is characterized as hospital-acquired MRSA (HA-MRSA) or community-acquired MRSA (CA-MRSA) (Table 1).

**Table 1:** The differences between HA-MRSA and CA-MRSA. Source: [4,5]. Note: This table is the author's creation and has not been reproduced from any published source.

Characteristics	Hospital-acquired MRSA (HA-MRSA)	Community-acquired MRSA (CA-MRSA)
Demographic	Found in patients with prior hospitalization	Found in specific populations
Associated infections	Nosocomial infections (e.g., endocarditis)	Causes skin and soft tissue infections
Phenotypic differences	Less virulent strains	Newer, more virulent strains
Staphylococcal cassette chromosome mec types	I, II, or III	IV or V
Panton-valentine leucocidin encoding genes	Not present	LukS-PV and LukF-PV
Antimicrobial susceptibility	Often resistant to aminoglycosides	Susceptible to non-β-lactam antimicrobials
Prevalence in hospitals	Coexisting with CA-MRSA	Predicted to replace HA-MRSA

It is pivotal to note that both strains are resistant to several medications like macrolides, tetracyclines, and lincosamides [4]. Burn patients are particularly susceptible to HA-MRSA due to the loss of the skin barrier, longer hospital stays, and immunodeficiency [1]. Burn intensive care units (BICUs) have higher rates of primary bloodstream infections (BSIs) compared to other ICUs. Despite rigorous standard-of-care practices, burn units may still experience high rates of MRSA transmission and MRSA bacteremia. Implementing infection control bundles, such as bed rotation, ultraviolet light-C (UVC) light disinfection, replacement of wound carts, and chlorhexidine surgical scrub, can significantly reduce MRSA colonization and bacteremia in high-risk burn populations [6].

MRSA is a significant cause of bacteremia, leading to 12% of cases of endocarditis [5], with central venous catheters and pneumonia being the most common sources of infection [7]. Pneumonia is a common infection that occurs in hospitals. Signs of the illness include coughing, difficulty breathing, fever, fast breathing, low blood pressure, and mental confusion [8]. Patients who are more susceptible to infection are more vulnerable to MRSA transmission through hand-to-hand contact with colonized patients or healthcare personnel [9]. Patients undergoing liver transplants and using prosthetic devices are at a higher risk of MRSA infection [10]. Nosocomial transmission of MRSA is accelerated by several factors, including poor compliance with surveillance cultures, a lack of adherence to contamination precautions, and poor hand hygiene [11].

Healthcare workers should use infection control measures to prevent the spread of MRSA [10]. Promptly diagnosing and isolating patients, particularly those in burn units and intensive care settings, is crucial due to the potential for MRSA transmission between individuals [12]. Strategies to control MRSA in burn patients include isolation, personal protective equipment (PPE), regular disinfection, and prophylactic intranasal mupirocin [7]. Implementing infection prevention and control measures, such as testing upon admission, contact precautions (CPs), co-locating infected patients, and promoting hand sanitizer use, effectively reduces the spread of MRSA and vancomycin-resistant enterococci (VRE) [13]. Early incision and grafting can prevent infection in burn wounds. Older burn patients with total body surface area (TBSA) >10% and inhalation injury benefit from a strict prevention plan [8]. MRSA can infect most equipment, such as catheters, surgical instruments, respiratory equipment, and even wound dressings in ICUs, so regular disinfection is necessary [11]. Eliminating endogenous and exogenous sources of *S. aureus* is important to prevent the colonization of burn wounds [9]. Confinement of MRSA-positive patients in separate divisions has two benefits: it protects other patients from exposure and emphasizes the need for precautions [14]. Evidence-based recommendations suggest hospitals with MRSA should check healthcare workers.

## Review

Healthcare-associated infections and MRSA

Healthcare-associated infections (HCAIs) are common in hospital settings, especially in burn units. Methicillin-resistant *Staphylococcus aureus* (MRSA) is the most commonly associated pathogen with HCAIs. Among the pathogenic characteristics of MRSA are staphyloxanthin, which increases pathogenicity by inactivating the microbicidal effect of superoxide; toxic shock syndrome toxin (TSST), which causes toxic shock; exfoliatin, which causes skin scalding; and alpha-toxin, which kills cells. Infections caused by MRSA can cause systemic malaise in addition to boils, inflammation, and pustules [15].

Transmission of MRSA in a hospital setting

The prevalence of MRSA infections among burn patients is not well documented. Most studies have focused on the proportion of MRSA in general ICUs, and there is no global assessment of MRSA infections in burn ICUs. This is a significant gap in the literature, as burn patients are at an increased risk of infection. Understanding the extent of MRSA infections in burn patients is essential for developing effective strategies to prevent transmission, infection, and mortality in this vulnerable population. However, a review by Khan et al. found that the MRSA infection prevalence among burn patients admitted to the burn ICU stands at 55.0%. The elevated occurrence of MRSA-related bloodstream infections within this particular burn center can be attributed to several factors, including the severe nature of the injuries sustained by admitted patients, early surgical removal of dead tissue (necrotomies), the initiation of enteral feeding, prolonged hospital stays, and extensive antibiotic treatments [16]. Burn patients are more vulnerable to colonization and infection by nosocomial bacteria due to the destruction of their skin's protective barrier and reduced immunity [15]. In these patients, the compromised skin barrier from burn wounds allows MRSA bacteria to infiltrate the body. Direct contact with contaminated burn wounds, often occurring during dressing changes or medical procedures, can lead to transmission. Thus, skin-to-skin and skin-to-fomite interactions are the most common routes.

In the intensive care unit (ICU) setting, the potential for MRSA transmission is elevated due to the critical condition of patients and the invasive procedures they undergo. Healthcare workers play a pivotal role in transmission, as contaminated hands can introduce MRSA to patients during medical interventions or routine care activities. The ICU environment presents risks, with medical equipment and frequently touched surfaces serving as reservoirs. Patient-to-patient transmission is facilitated by proximity and shared spaces, leading to direct or indirect contact. Invasive procedures common in ICUs, such as catheter insertions, mechanical ventilation, and central line placements, offer opportunities for MRSA to breach the body's defenses. The presence of wounds, surgical sites, and invasive lines further heightens vulnerability to MRSA infection among ICU patients [17]. 

Trends and factors of MRSA infections

Studies conducted in low-income countries have reported a notably high incidence of MRSA infections. This elevated occurrence could potentially be linked to the irrational use of antibiotics, the substandard hygiene conditions often found in healthcare facilities, as well as the prevalence of patients from lower socioeconomic backgrounds who frequently encounter issues related to inadequate sanitation, malnutrition, and subpar personal hygiene practices. Furthermore, the heightened risk of *S. aureus *infections in this patient group is compounded by the close quarters in which patients with severe medical conditions and healthcare workers often interact. In contrast, lower MRSA incidence rates observed in developed countries can be attributed to proactive surveillance and the treatment of MRSA cases. In these regions, routine screening of both hospitalized patients and healthcare workers for MRSA has shown promising outcomes [16]. In the United States, the incidence of healthcare-associated MRSA bloodstream infections (HA-MRSA BSIs) declined by 74% from 2005 to 2016. This could be largely attributed to improvements in infection control practices such as hand hygiene, contact precautions, and environmental cleaning; newer antibiotics, such as linezolid and daptomycin, effective against MRSA; and the emergence of community-associated MRSA (CA-MRSA). However, the rate of decline in MRSA BSIs has slowed in recent years, and CA-MRSA infections have increased. USA300 is the most common clone of MRSA in the United States, and it is responsible for a large number of infections. MRSA CC398 is a newer clone that is becoming increasingly common, and it is also more resistant to antibiotics than USA300. This may be due to a number of factors, including the overuse/misuse of community-acquired antibiotics; the increasing number of people with chronic health conditions such as diabetes and human immunodeficiency virus (HIV) that lead them susceptible to MRSA; the globalization of travel that causes people to be more likely exposed to MRSA and to bring it back to their home countries [18]. A similar trend of declining prevalence of HA-MRSA and increasing CA-MRSA infections has also been observed across Europe and Australia [19,20].

In the pediatric setting, high colonization rates with CA-MRSA strains were observed [21]. This could be due to several factors: 1. CA-MRSA strains are becoming increasingly common. 2. Children are less likely than adults to be exposed to classic HA-MRSA strains. 3. Children are often in close contact with others in settings where cleanliness is limited, such as daycare, schools, and sports teams.

These settings have been identified as high-risk contexts for MRSA transmission [21] (Table 2). In a study of pediatric patients with severe *Staphylococcus aureus* infections admitted to the pediatric ICU, the most common reasons for ICU admission were respiratory failure requiring ventilation (71.4%) and septic shock (28.6%). Several children required multiple treatments, including ventilation for seven days, inotropic and vasopressor treatment for those who developed septic shock, and unilateral or bilateral pleural drains for those who developed pneumonia [22].

**Table 2:** Risk factors of MRSA acquisition. Source: [23-29]. Note: This table is the author's own creation and has not been reproduced from any published source. MRSA: methicillin-resistant *Staphylococcus aureus*, MSSA: methicillin-sensitive *S. aureus*.

Age group	Risk factors
Neonates	•Neonates come into contact with adult skin or their environment shortly after birth, where they are exposed to *Staphylococcus aureus* and can quickly get colonized. •Neonates are extremely likely to be exposed to *Staphylococcus aureus* during the first few weeks after birth because between 30 and 70% of people carry the bacteria. •The umbilical cord, skin, nasopharynx, and gastrointestinal system are the most typical sites of *S. aureus* colonization. •There is evidence to suggest that neonatal diarrhea, particularly in cases of exclusive breastfeeding, can be influenced by the presence of *S. aureus* on the mother's skin. Moreover, the vulnerability of the infant's gastrointestinal system due to potential nutritional deficiencies may further exacerbate this susceptibility. •Numerous studies have demonstrated a link between low birth weight and an increased risk of MRSA colonization and/or infection. Multiple gestations as well as prematurity and younger gestational ages have been proven to be risk factors for MRSA colonization and infection.
Children	The proportion of cases with leukocytosis was significantly higher in the MRSA than in MSSA followed by •Anemia, •Thrombocytopenia, •Septic shock, •Exanthematous illness, •Immunosuppressant usage.
Adults	•Recent hospitalization or antibiotic use, particularly hospitalization with receipt of IV antibiotics in the prior three months. •Recent viral pneumonia or Influenza infection can result in secondary bacterial pneumonia caused by MRSA. •Empyema caused by MRSA can be a complication in post-COVID-19 infection. •Necrotizing or cavitary pneumonia. •End-stage kidney disease. •Crowded living conditions (e.g., incarceration). •Injection drug use. •Contact sports participation. •Men who have sex with men.

Controversies surrounding contact precautions

Conversely, healthcare workers can also become infected with MRSA from patients. This can happen in the same way that MRSA can spread from patient to patient. Healthcare workers are at an increased risk of infection because they are in close contact with patients who may be infected with MRSA. To help prevent the spread of MRSA between patients and healthcare workers, it is vital to practice contact precautions (CP), such as: (i) Handwashing is the single most important way to prevent the spread of MRSA. Healthcare workers should wash their hands with soap and water for at least 20 seconds before and after contact with each patient. (ii) Using personal protective equipment: Healthcare workers should wear personal protective equipment (PPE) when caring for patients infected with MRSA. This includes gloves, gowns, and masks. (iii) Decontaminating surfaces: Surfaces that may be contaminated with MRSA should be decontaminated with an appropriate disinfectant. (iv) Educating patients and visitors: Patients and visitors should be educated about preventing the spread of MRSA. This includes washing their hands frequently and avoiding contact with the patient's wounds or bodily fluids [3].

Interestingly, a study by Morgan et al. argues that there is limited evidence to support the use of CP for the prevention of transmission of endemic MRSA and VRE and that CP can have several negative consequences, such as increasing the workload of healthcare workers; making it more difficult for patients to receive care; and contributing to the development of antibiotic resistance [22]. However, further research on this claim is warranted.

Controlling the spread of MRSA

MRSA is a pathogen that is prevalent in hospitals worldwide despite compliance with screening cultures [30]. Patients with a history of MRSA, frequent hospitalizations, and those in residential care facilities are at higher risk [26]. Even with good infection control programs, MRSA transmission continues due to the pooling effect of transferring patients from the ICU to burn units [31]. 

**Figure 1:**
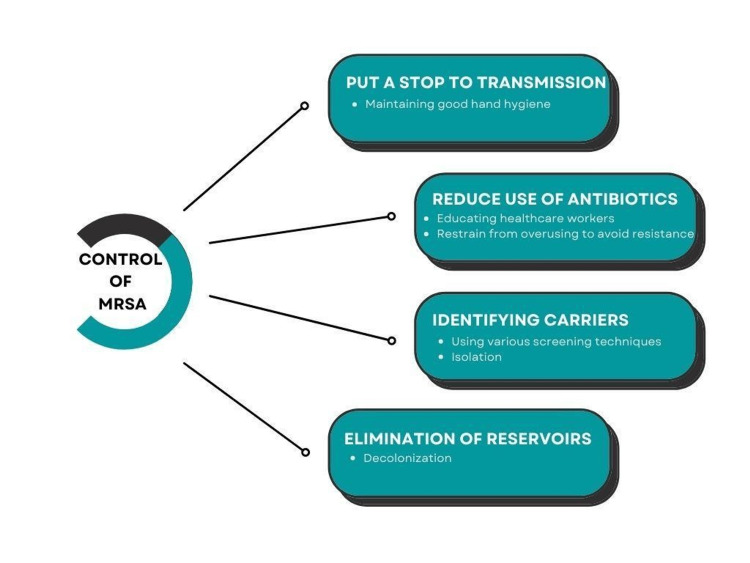
Strategies to control MRSA. This figure is the author's own creation and has not been reproduced from any published source. MRSA: methicillin-resistant *Staphylococcus aureus*.

As most transmissions in hospitals occur through the contaminated hands of healthcare workers, reliable and rapid detection of MRSA-colonized patients is crucial to reduce transmission [32]. Crucial strategies have been devised to control MRSA transmission, as shown in Figure 1. Standard care for burn patients involves accurate estimation of TBSA, fluid resuscitation, and the use of silver-based dressings [8]. Patients should undergo active surveillance cultures to screen for any infections, and those who are carriers should be isolated and treated with chlorhexidine gluconate (CHG) baths and mupirocin application to the nares for decolonization. Universal decolonization is more effective and less expensive than other strategies like individual screening and isolation. However, prolonged use of mupirocin could lead to resistance, reducing cost-effectiveness [33]. The most common methods of controlling MRSA involve judiciously using antibiotics, screening and isolating carriers, decolonization, and improving hand hygiene compliance [34].

Comprehensive eradication of colonization is pivotal in eliminating carriage and inhibiting transmission and infection. Coupled with strict infection control measures, this standardized multi-faceted approach to decolonization focuses on eliminating MRSA carriage from the primary nasal, skin, gastrointestinal, and genitourinary reservoirs [35].

**Figure 2:**
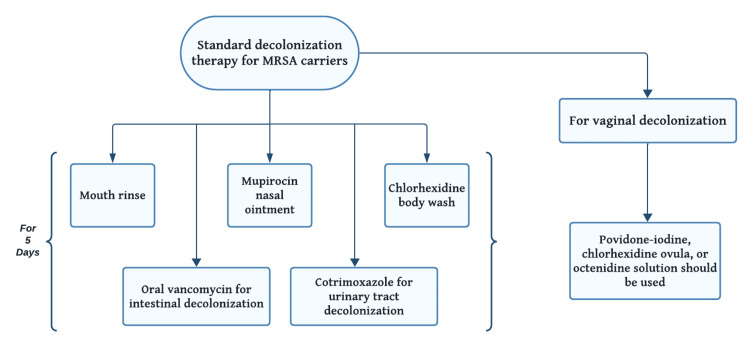
Decolonization therapy for MRSA. This figure is the author's own creation and has not been reproduced from any published source. MRSA: methicillin-resistant *Staphylococcus aureus*.

Disposable daytime clippers can be used for hair removal and antiseptic solutions and are recommended for incision site preparation [31]. Standard decolonization therapy for MRSA carriers involves using topical and systemic antimicrobials to eliminate nasal, skin, gastrointestinal, and genitourinary tract colonization. Further explanation is below in Figure 2.

Most MRSA carriers are asymptomatic, active surveillance or screening using screening cultures or rapid nucleic acid amplification tests (NAATs) may be carried out to detect MRSA. Healthcare facilities should tailor their testing strategy to the local epidemiology of MRSA. Caution should be exercised with widespread chlorhexidine bathing, as there are issues with allergy, intolerance, and increased costs. Several surfaces can become contaminated with MRSA, and a colonized patient can continue to transmit MRSA even after leaving the hospital [36].

MRSA control programs include active surveillance and isolation of individuals with MRSA carriage [37]. Screening allows for the monitoring of MRSA genotypes and local antibiotic resistance patterns. Antimicrobial susceptibility patterns and variants of those patterns serve as valuable tools for analyzing local antibiotic resistance patterns and treatment options [13]. Contact precautions are recommended in acute care settings for patients with MRSA infections or colonization, while the use of personal equipment (gowns/gloves) can reduce infection rates. As part of decolonization, MRSA carriage is suppressed or eradicated to reduce transmission within an institution, as well as infection risks for the colonized patient [36].

Mupirocin is the most effective treatment for nasal MRSA carriage, with a success rate of up to 90% [36]. Preoperative hospital screening for MRSA is ambiguous and should be used as a preventive measure in patients with a high risk of infection [38]. A significant reduction in nosocomial MRSA infections was seen in hospitals following the MRSA screening and decolonization protocol [39].

Strategies for implementing MRSA reduction practices

**Figure 3:**
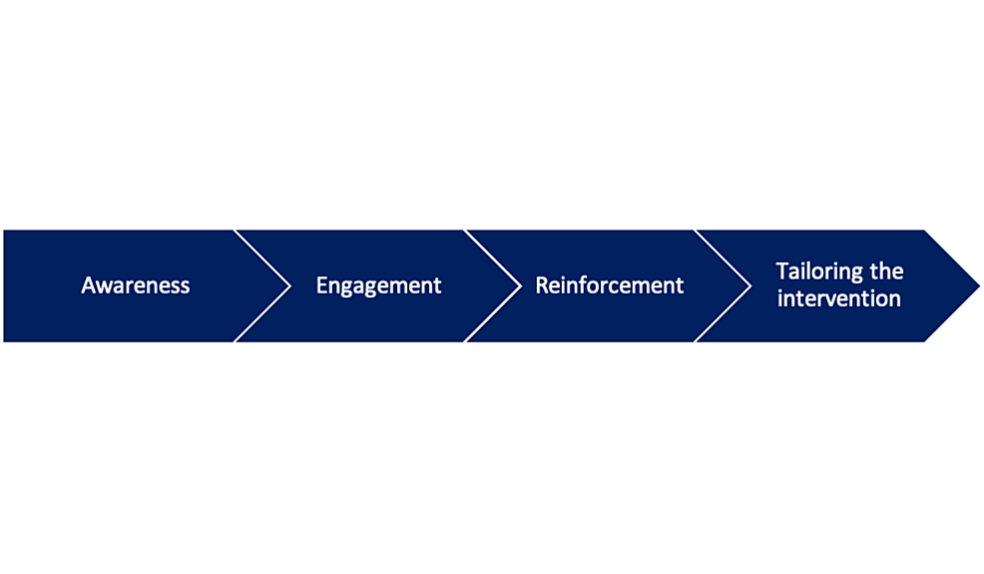
Implementation activities for MRSA reduction. This figure is the author's own creation and has not been reproduced from any published source. MRSA: methicillin-resistant *Staphylococcus aureus.*

In comparison to methicillin-sensitive *S. aureus* (MSSA), the mortality, length of stay, and cost of healthcare associated with MRSA are likely higher due to the pathogen's virulence and survival fitness [40]. Implementation activities have been targeted under multiple interventions to control MRSA, as shown in Figure 3 [41].

As part of the implementation activities, awareness of MRSA reduction strategies and the frequency, severity, and effects of MRSA infections on patients and staff was raised in a variety of methods [41]. 

In order to successfully control MRSA infection, it is crucial to actively engage and motivate frontline employees as vital contributors to the MRSA reduction initiative. Additionally, enlisting credible clinicians can help garner attention and support for the project team [42]. Offering feedback to frontline employees is a proven approach as it provides concrete proof that the implemented strategies are effective, as reductions in MRSA rates can be observed. Maintaining a positive outlook and fostering awareness of staff and team empowerment are valuable approaches to support change and sustain momentum in the project [42]. Adaptation to local systems, whether at the unit, floor, or hospital level, is crucial for successful intervention. Flexibility and engaging frontline employees in shaping and adapting the local implementation plan, even when MRSA strategies remain consistent across participating systems, can yield substantial benefits in the management of MRSA infections [42].

Effective control of MRSA transmission hinges on maintaining and adhering to stringent hand hygiene practices [43]. MRSA transmission plays a crucial role in environmental contamination and is frequently observed in the close vicinity of colonized patients [44,45]. MRSA can survive on surfaces for months as a reservoir for transmission [46].

Cost-effectiveness and challenges faced in the implementation of prevention strategies

Although many measures have been evaluated for their effectiveness in reducing hospital-acquired MRSA, there is still much debate on which infection management practices are the most successful. Significant debate persists regarding the importance of different treatments, primarily due to their high costs, potential interference with patient care, and the challenge of preventing endemic transmission [47].

Implementation of prevention strategies has many challenges. For instance, in small hospitals active screening may not be possible due to the lack of staff in infection control programs and fewer funds and lack of capability for MRSA screening [48]. Qualitative research conducted by Seibert et al. revealed that numerous healthcare professionals encountered challenges when it came to consistently practicing regular hand hygiene and adhering to contact precautions. Patient care needs, equipment, and environmental difficulties such as sink availability, time constraints, the behaviors of other healthcare workers, and the requirement for extra signs identifying which patients require contact precautions were some challenges that were identified on a functional level [49].

MRSA continues to pose a substantial health threat in Europe, characterized by elevated infection rates in certain countries and a range of challenges in its containment. These challenges include inadequate infection control measures, resource limitations, and the emergence of new MRSA strains [50]. MRSA costs hospitals and healthcare systems money, yet screening programs for MRSA have been demonstrated to be cost-effective not just with traditional culture-based detection methods but also with the more expensive PCR-based detection [50]. In a study by Gidengil et al., the efficiency and costs of four different methods for preventing MRSA transmission and infection in the ICU were compared: standard infection control practices, screening and isolating MRSA-positive patients, decolonizing MRSA carriers, and a combination of screening, isolation, and decolonization. The research found that the combination approach was the most effective in reducing MRSA transmission and infection in the ICU, but it was also the most expensive [33].

The screening and isolation strategy was less effective than the combination strategy but more cost-effective than the decolonization strategy. The researchers concluded that while a combination of screening, isolation, and decolonization might be the most efficient way to prevent MRSA transmission and infection in an ICU, the decision to adopt this strategy should also take into account its additional costs [33]. A study conducted by Win et al. assessed a two-year screening initiative aimed at preventing the spread of methicillin-resistant *Staphylococcus aureus* (MRSA) within healthcare facilities in Singapore. This program involved screening patients admitted to acute care hospitals and providing targeted interventions to those identified as having MRSA colonization [51]. Additionally, the study evaluated the cost-effectiveness of the program, considering expenses related to screening, interventions, and treating MRSA infections. The authors determined that the program represented a cost-effective strategy for reducing MRSA infections, with an incremental cost-effectiveness ratio (ICER) of SGD 11,375 per quality-adjusted life year (QALY) gained [51]. In summary, the authors concluded that MRSA screening programs constitute a valuable investment in mitigating MRSA infections within healthcare settings in Singapore [51].

## Conclusions

This narrative review has shed light on the significant concern of MRSA colonization within intensive care and burn units. MRSA, a formidable antibiotic-resistant pathogen, poses a considerable threat to vulnerable patients in these settings. The review has highlighted the multifaceted factors contributing to MRSA colonization, including patient susceptibility due to compromised immunity, invasive medical interventions, and environmental influences. Moreover, this review underscores the critical role of healthcare workers as both vectors and gatekeepers of MRSA transmission. Efforts to combat MRSA colonization demand a comprehensive approach, encompassing stringent infection control protocols, surveillance, and early detection. Education of healthcare personnel and patients alike, along with judicious antibiotic use, emerges as pivotal in this battle.

As we move forward, it is imperative that healthcare systems and providers remain vigilant in the fight against MRSA colonization, implementing evidence-based strategies to protect the most susceptible among us. Continued research and international collaboration will further our understanding and ability to control this persistent threat in intensive care and burn units, ultimately improving patient outcomes and reducing the burden of MRSA-related infections.
